# Neuroregulatory role of ginkgolides

**DOI:** 10.1007/s11033-021-06535-2

**Published:** 2021-07-10

**Authors:** Martyna Gachowska, Wojciech Szlasa, Jolanta Saczko, Julita Kulbacka

**Affiliations:** 1 Faculty of Medicine, Wroclaw Medical University, Wrocław, Poland; 2 Department of Molecular and Cellular Biology, Faculty of Pharmacy, Wroclaw Medical University, Wrocław, Poland

**Keywords:** Ginkgolides, *Ginkgo biloba*, Neuro-regulation, Neuroprotection

## Abstract

The application of ginkgolides as a herbal remedy reaches ancient China. Over time many studies confirmed the neuroprotective effect of standard *Ginkgo biloba* tree extract—the only available ginkgolide source. Ginkgolides present a wide variety of neuroregulatory properties, commonly used in the therapy process of common diseases, such as Alzheimer’s, Parkinson’s, and many other CNS-related diseases and disorders. The neuroregulative properties of ginkgolides include the conditioning of neurotransmitters action, e.g., glutamate or dopamine. Besides, natural compounds induce the inhibition of platelet-activating factors (PAF). Furthermore, ginkgolides influence the inflammatory process. This review focuses on the role of ginkgolides as neurotransmitters or neuromodulators and overviews their impact on the organism at the molecular, cellular, and physiological levels. The clinical application of ginkgolides is discussed as well.

## Introduction

### Ginkgolides

**Fig. 1:**
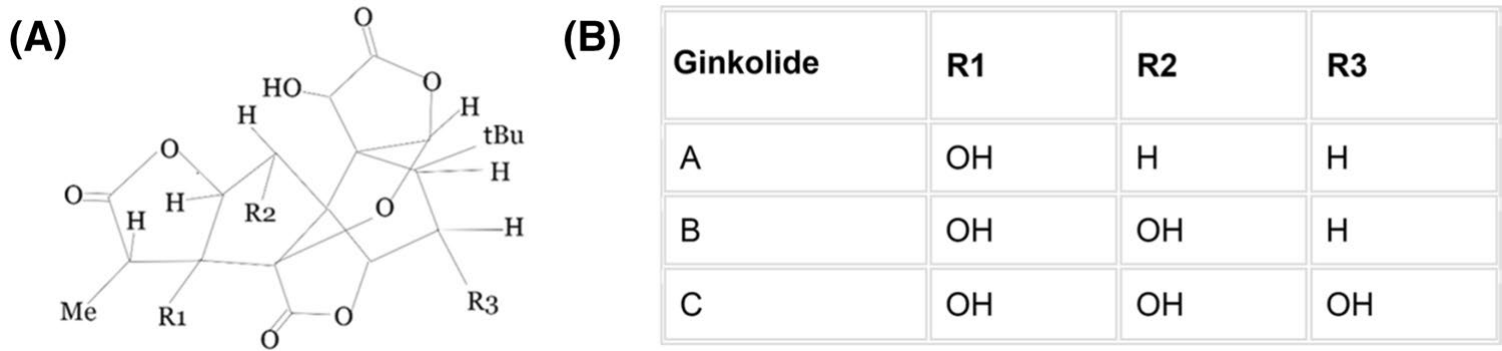
Ginkgolides classification; **A** General chemical structure of ginkgolides; **B** Classification of ginkgolides based on the substituent to the core structure; based on S. Omar “Ginkgolides and Neuroprotective Effects”

Ginkgolides belong to the group of terpene trilactones (TTLs) with a unique chemical structure. Ginkgolides are isolated from one of the oldest tree species—*Ginkgo biloba* tree—the only remaining species of the Ginkoales [1]. Besides ginkgolides, the *G. biloba* tree is a source of flavonoids. The standardized extract from its leaves—EGb 761, contains 24% flavonoids (mainly flavonol O-glycosides, quercetin, kaempferol, isorhamnetin, and proanthocyanidins) and 6% of terpenoids (which include 2.8–3.4% of ginkgolides and 2.6–3.2% of bilobalide). So far, *G. biloba* is the only known source of ginkgolide A, B, C, M, J, and K. All of the mentioned ginkgolides are characterized by the system of five-membered rings—one spiro-noncarocylic, three lactones, and a tetrahydrofuran ring. Tert-butyl group is also present in the core structure. Ginkgolides can be divided into different types according to their substituents [2] (Fig. 1).

Besides the beneficial role of ginkgolides on health and well-being, some acidic derivatives tend to develop an allergic reaction after the administration. However, standard medical product EGb 761 presents none of them. Table 1 presents the characteristics of individual ginkgolides, demonstrating the possible function and biological effects. Further, there will be an overview of each of the following traits in the neuroregulatory properties of ginkgolides.

**Table 1:** Functions of ginkgolides; based on S. Omar “Ginkgolides and Neuroprotective Effects”

Function	Ginkgolide	Effect
Anti-inflammatory	A, B, C, J	IL-5 and IL-13 inhibition MAPK (mitogen-activated protein kinase) inhibition TLR (toll-like receptor) inhibition Inhibitor of arachidonic acid inflammatory pathway
Anti-coagulant	B	PAF (platelet-activating factor) inhibition
Anti-oxidative	A, B	NF-kB (Nuclear factor kappa B) inhibition
Anti-apoptotic	A, C, J, M, K	TNF receptor inhibition
Anti-oxidative	B, C, J, M	Free radical scavengers

This review paper discusses the role of ginkgolides in neuroregulation and immunomodulation. Further, the authors present the interaction between ginkgolides and various receptors. In the main part of the review, the reader is introduced to the impact of ginkgolides on the neurotransmitters and main transmitter pathways. In the end, the paper focuses on the clinical application of natural drugs.

### Neuroregulators

Neuroregulation plays a crucial role in the coordination of nervous system activity [3]. Indeed, neuroregulators regulate the communication between the neural system cells. Considering the function of neuroregulators, they can be divided into neurotransmitters and neuromodulators.

Neurotransmitters are substances synthesized and secreted in nervous system cells. After the chemical synapse secretion, the compounds might bind both to specific post- and presynaptic receptors. Conversely, there could be distinguished ionotropic and metabotropic neuroreceptors. By binding to the ion channels, the neurotransmitters induce direct opening of the channel’s pore and the transmission of ions—leading to excitatory or inhibitory postsynaptic potential passage. Neurotransmitters convey a transient and unilateral signal only across the specialized synapse. On the other hand, metabotropic neurotransmitters affect the neuronal cell's metabolic activity by the G-protein related to the cellular signaling pathway [3].

In contrast to neurotransmitters, neuromodulators influence the neuronal tissue outside as well as in the synapse. The compounds usually bind to the metabotropic receptors. Neuromodulators activate secondary transmitters, therefore modulating the sensitivity of the cell towards other neurotransmitters. Furthermore, the neuromodulators can alter neuronal signal transmission by controlling the number of neurotransmitters synthesized and released from the neurons. Contrary to neurotransmitters, neuromodulators coordinate a long-lasting response due to the lack of reabsorption by pre-synaptic neurons or metabolic degradation [4].

Some neuroactive substances can be classified as neurotransmitters as well as neuromodulators, e.g., acetylcholine. The impact of neurotransmitters on cells varies depending on the receptor that interacts with acetylcholine. The development of selective drugs depends on the proper characteristics of the interactions between the ligand and its receptor [5]. The nature of neuroregulators and neurotransmitters was summarized in Table 2.

**Table 2:** The comparison between neurotransmitters and neuromodulators; based on Konturek S. J. Human’s Physiology

	Neurotransmitters	Neuromodulators
Most of the effect on	Postsynaptic neuron	Multiple neurons
Reabsorption	Yes	No
Time of action	Short	Long
Main receptors	Ionotropic receptors	Metabotropic receptors

## Immunomodulatory role of ginkgolides

### Ginkgolides as PAF inhibitors in inflammation and thrombotic process

The inflammatory response is a complex process in which the immune system reacts to potentially harmful stimuli, such as pathogens, toxins, or damaged cells [6]. One of the inflammatory mediators is a PAF (platelet-activating factor, 1-O-alkyl-2-acetyl-sn-glycero-3-phosphocholine) [7]. PAF is responsible for the initiation and progression of inflammatory and thrombotic reactions. PAF recruits and activates leukocytes, boosts the production of cytokines and chemokines, and induces angiogenesis. The cytokine targets cells via a specific G-protein-coupled metabotropic receptor (PAFR). The interaction between PAF and PAFR induces signal transmission in the humoral, autocrine, or paracrine way [8]. Activated PAFR triggers various intracellular signal cascades, including turnover of phosphatidylinositol, elevation in intracellular calcium concentration, and activation of kinases [9]. Phosphatidylinositol is a lipid that takes part in cell signaling [10]. Its degradation leads to the release of PIP3 (Inositol Triphosphate) and DAG (Diacylglycerol). Both of the substances are involved in long-lasting cell responses [11].

PAF is synthesized both in acute and chronic inflammation response types. Thus, by the blockage PAFR, the inhibition of inflammation and ischemic injury might occur. The process provides the interaction of ginkgolides and PAFR, thus locking PAFR in its inactive state [12]. Ginkgolides and PAF are competitive antagonists. As the neuromodulators, ginkgolides bind to the G-protein-coupled receptors themselves. In the case of PAF-modulatory effects, Ginkgolide B is the most potent antagonist of the PAFR. Besides, it was proved that Ginkgolide B interrupts leukocyte activation in inflammation. In vivo experiments showed that, in PAF-induced conditions, ginkgolide B interrupts leukocyte activation. It constricts leukocyte locomotion by inhibiting chemotaxis. It also inhibits the binding of PAF to eosinophils and neutrophils [13].

### Ginkgolides as TLR4 antagonists

Ginkgolides are responsible for the inhibition of TLR4-mediated inflammatory response [8, 14] . After binding to TLR agonists, such as bacterial or viral products, TLRs activate the adapter proteins (MYD88, TRAM, TIRAP), eventually activating the phosphorylation of NF-kB (nucleus factor kappa B) [15]. Ginkgolides are also responsible for activating JAK2/STAT3 (Janus kinase 2/signal transducer and activator of transcription 3) and p38 MAPK (mitogen-activated protein kinase) pathways, further affecting the immune system cells [16].

### Ginkgolides as PLA2 inhibitors

Another platelet-related role of ginkgolides is the inhibition of PLA2 (phospholipase A2). When ginkgolide, as a ligand, binds to the G-protein-coupled receptor, it activates the adenyl cyclase, and consequently, the cAMP level increases. Further, cAMP activates protein kinase C leading to the activation of PLA2, which releases arachidonic acid (AA) from membrane phospholipids, and AA is also the precursor of eicosanoids, e.g., leukotrienes and prostanoids. PLA2 is engaged in the first stage of arachidonic acid synthesis [17]. When ginkgolide, as a ligand, binds to the G-protein-coupled receptor, it stops the activation of the adenyl cyclase, and the cAMP level decreases, leading to decreased level of arachidonic acid [18].

## The influence of ginkgolides on neurotransmitters

### Influence of Ginkgolides on glutamate transmission

Ginkgolides modulate glutamate transmission—the major excitatory neurotransmitter of the cortex and hippocampus. There are two main types of glutamate receptors distinguished: ionotropic receptors and G-protein coupled, metabotropic receptors. There are three types of ionotropic receptors, four subunit tetramers: (1) N-methyl-D-aspartate (NMDA), (2) α-amino-3-hydroxy-5-methyl-4-isoxazole propionic acid (AMPA), and (3) kainate receptors (KA). The binding of glutamate to the receptors activates a short-time modulation pathway by allowing for the flow of ions inside or outside the cell. In opposite, activating the metabotropic receptor starts the long-lasting neuronal modulation. The flow of calcium ions triggers the secretion of glutamate [19]. As was previously mentioned, ginkgolides influence the PLA2 and prevent activation of kinase C. Kinase C has a great impact on the circulation of calcium because it stimulates DAG (diacylglycerol) and IP3 (inositol triphosphate), responsible, e.g., realize of calcium from the endoplasmic reticulum. When the pathway is inhibited, calcium flow is stopped, and glutamate cannot be released to the synapse. Ginkgolides' inhibitory function is applied in the attenuation of the glutamate-induced damage of neuronal hippocampal cells and cerebral ischemia. Furthermore, Alzheimer’s Disease studies proved that ginkgolides inhibit both NMDAR (*N*-methyl-D-aspartate receptor) and AMPAR (α-amino-3-hydroxy-5-methyl-4-isoxazole propionic acid receptor. Bilobalide, which derives from ginkgolides, inhibits NMDAR by affecting the glycine spot independent from strychnine [20]. Consequently, the NMDA receptor requires glutamate and glycine as well as ions of magnesium to activate. Accordingly, ginkgolides A, B, C, and J were classified as blockers of glycine-activated receptors [21]. Ginkgolides were proved to block the open chloride channel of glycine-activated receptors. Further research demonstrated that the effect of ginkgolides depends on their concentration [22]. Ginkgolides act as non-competitive antagonists towards glycine-activated receptors. Ionotropic receptors are more common to bind with neurotransmitters.

### Influence of ginkgolides on γ-aminobutyric acid

**Fig. 2:**
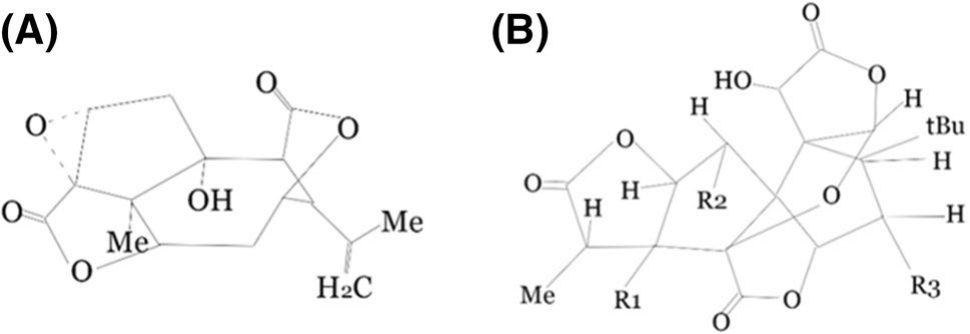
The comparison between the chemical structure of **A** picrotoxin and **B** ginkgolide; based on Huang SH, Lewis, et al. Mixed antagonistic effects of the ginkgolides at recombinant human ρ1 GABAC receptors

It is known that glutamate receptors are related to the GABAA (γ-aminobutyric A) receptors [23]. Both of these channels are ion channels, selectively permeable to the chloride anion. Namely, both receptors are considered to be ionotropic receptors. γ-aminobutyric acid (GABA) is the primary inhibitory neurotransmitter in the brain and the spinal cord. It is synthesized in a presynaptic neuron from the glutamate precursor. GABA receptors are divided into two groups. One of them includes GABA A and GABA C receptors, postsynaptic ionotropic receptors responsible for cell hyperpolarisation [24]. The structures belong to the pentametric Cys-loop ligand-gated ion channel superfamily (Gly receptors, 5-HT3 receptors, and nACTH receptors) [25]. GABA A and GABA C receptors differ in sensitivity to, e.g., Bicuculline (receptor antagonist). In this case, hyperpolarisation develops due to the increased conductance of chloride ions. It inhibits the conduction of the action potential. The second group consists of metabotropic GABA B receptors. Via the G protein-coupled receptors, the conductance of potassium increases, opposite to decreasing calcium conductance. There are few well-known GABA receptor blockers. One of them is picrotoxin (PTX). The chemical structure of PTX and ginkgolides is highly similar. The two substances have both oxygenated chain-like structures and lipophilic side chains (Fig. 2).

Ginkgolides and PTX have a similar pocket of binding at GABA and glycine receptors. Although PTX is toxic to humans, ginkgolides are used as herbal medicines. This influence is somewhat surprising because most GABA inhibitors are defined as pro-convulsant. Research is still in progress to explain the effect. PTX and ginkgolides bind at the 2′ or/and 6′ pore-lining position to the chloride ion channels. Here, ginkgolides are considered as mixed non-competitive antagonists of chloride channels [26]. Considerable neuromodulators influence the majority of metabotropic receptors. Finally, the impact of ginkgolides on GABA A and GABA C receptors is much more prominent than on GABA B receptors.

### Ginkgolides’ influence on serotoninergic transmission

5-HT3 receptor, defined as one of the members of the Cys-loop ligand-gated ion channel superfamily, is also one of the serotonin receptors. 5-HT3 receptors are divided into homopentameric receptors—5-HT3A and heteropentameric receptors – 5-HT3B. Both of the receptor groups allow for the flow of sodium or potassium ions to the cell. Homopentameric channels are also permeable for calcium ions and small cations. Like in the last paragraph, similarly to ginkgolides—PTX is also an antagonist of 5-HT3 receptors. Due to the structural similarity of ginkgolides to PTX, the influence of ginkgolides on serotoninergic transmission is reasonable. Indeed, ginkgolides also act on cation-selective receptors. Both PTX and Ginkgolides block the 5-HT3 by binding in a similar location—the structures can be described as competitive antagonists. Moreover, ginkgolides have different influences on homomeric and heteromeric 5-HT receptors, depending on the concentration of ligand [27].

### Ginkgolides’ influence on dopaminergic and noradrenergic transmission

Dopamine (DA) is a neurotransmitter synthesized in the substantia nigra, ventral tegmental area, and hypothalamus of the brain. DA is involved in the modulation of cognition, voluntary movements, punishment, and reward [28]. DA is also a precursor of epinephrine and norepinephrine (NE). DA receptors include postsynaptic and presynaptic receptors. Regardless of the receptor, the interaction triggers electric potential in the presynaptic cell [29]. The potential may be both excitatory (postsynaptic and presynaptic receptors) or inhibitory (presynaptic receptors). Clinical data suggests that ginkgolides improve cognitive function. Kehr showed in 2010 that chronic administration of ginkgolides results in an increased dopaminergic and noradrenergic transmission in the frontal cortical brain area [30]. Furthermore, Ginkgolides increase dopaminergic activity in the paraventricular nucleus in rats [31]. This effect may be explained by the inhibitory effect of ginkgolides on MAO (monoamine oxidase). MAO is responsible for disposing of norepinephrine in a synapse. Yoshaitake et al. proved that a single oral dose did not affect monoamine concentration levels [30].

Chronic administration of EGB 671 did not affect MAO activity in homogenates of mice brains. The extracellular level of dopamine was much higher than the noradrenaline level. This differs from the MAO inhibitor effect, which results in a similar concentration of the monoamines in extracellular fluid even after one injection. A possible explanation of this effect is the desensitization or down-regulation of DA and NE receptors induced by the ginkgolides’ admission. Desensitization is a classic way of neuromodulation. It is manifested by the bond between the receptor and neuromodulator, leading to the reduction of the receptor’s responsiveness.

## Ginkgolides neuromodulatory effect

### Effects on apoptosis

Ginkgolides are responsible for neuroprotection. Ginkgolides reduce apoptosis in rat brain tissue cells with acute cerebral infarction, thus protecting the brain cells [27]. Apoptosis is defined as programmed cell death, executed by caspases. It may occur extrinsic—by death receptor pathway and intrinsic—by the mitochondrial pathway. The TNF (tumor necrosis factor) receptor ligands are the main inducers of apoptosis in the central nervous system (CNS). Two types of tumor necrosis factor receptors (TNFR) are distinguished: TNFR1 and TNFR2. Both lead to effector caspases activation. TNFR are transmembrane proteins coupled with adapter proteins. The anti-apoptotic role of ginkgolides is also considered when analyzing the effects of ginkgolides [32]. Although further research still has to be done, already collected data show that: (1) ginkgolides inhibit the mRNA transcription responsible for caspase expression, (2) weaken mRNA for Bax expression, consequently leading to the decreased caspase 9 activity and change in mitochondrial membrane potential (MMP). Ginkgolides might be considered as inhibitors of the death receptors or may downregulate the receptor by desensitization.

The available data revealed that the anti-apoptotic function of ginkgolides towards normal cells, especially ginkgolide B, stays in contrast to the induced apoptosis in MCF-7 breast cancer cells in in vitro experiments. The studies demonstrated that treatment with ginkgolide B induces apoptotic biochemical changes as calcium ions inflow, production, loss of MMP, and increase p53 and p21 expression [33]. These opposite effects depend both on the type of ginkgolide as well as on the type of cell.

### Ginkgolides as free radical scavengers

The oxidation and reduction process occurs in each type of cell. This process leads to the production of reactive oxygen and nitrogen species (RONS). RONS may also be produced in mitochondrial electron transport. Each cell developed various defensive mechanisms to prevent the harmful effect, including the inversion of oxyhemoglobin into methemoglobin, peroxidation of lipids, brain ischemia, and damage in proteins resulting from oxidative stress [34]. Numerous scientific papers show the antioxidant activity of ginkgolides. In vitro, ginkgolides of EGB 761 act as free radical scavengers via NADPH-oxidase inhibition (NADPH is necessary as reduction equivalent donor) and blocking Aβ-induced events such as reactive oxygen species accumulation and apoptosis [35]. Moreover, ginkgolides upregulate antioxidant proteins' levels by mediating the Akt/Nrf2 signaling pathway to protect neurons from oxidative stress injury [36]. Ginkgolides B, C, J, M react with superoxide, which also proves their antioxidant properties. Only ginkgolide A does not react with superoxide in these trials [37]. All the biochemical effects of ginkgolides were summarized in Table 3.

**Table 3:** The action of ginkgolides assigns to the type of neuroregulator

Action	Neurotransmitter	Neuromodulator	References
Ginkgolides as PAF inhibitors: binding to metabotropic receptors		+	[8]
Ginkgolides as TLR4 antagonists: long-lasting regulation		+	[16]
Ginkgolides as PLA2 inhibitors: neuronal signal modulation	+	+	[38]
Ginkgolides vs glutamate: NMDA and AMPA inhibition (ionotropic receptors)	+		[20]
Ginkgolides vs GABA: Binding to GABAA and GABAC receptors (ionotropic receptors)	+		[26]
Ginkgolides influence on serotonin transmission: binding to the 5-TH3 receptors (ionotropic receptors)	+		[27]
Ginkgolides vs dopamine and noradrenaline: desensitization		+	[30]
Ginkgolides vs apoptosis: TNFR inhibition and desensitization		+	[32]
Ginkgolides as free radical scavengers: the oxidative proteins level upregulation		+	[36]

## Therapeutic role of ginkgolides

The therapeutic role of ginkgolides was proved in various diseases, including neurodegenerative disorders, cancer, hepatic diseases, and widely used as an anti-inflammatory agent [39].

### Acute pancreatitis

Acute pancreatitis is a leading gastrointestinal disorder, which untreated leads to death. It is manifested by pancreas inflammation, systemic inflammatory response syndrome, and multiple organs dysfunction syndrome. It is caused by the damage of acinar cells. Acinar cells are responsible for the synthesis, storage, and secretion of digestive enzymes production in the pancreas [40]. The mechanism involves PAF-mediated damage of the acinar cells. As ginkgolides were proved to be the PAF inhibitors, their administration reduced necrotic and inflammatory changes in pancreatic tissue [13]. PAF competitive inhibitors blocks PAFR in its inactive state. Ginkgolides prevent phospholipase C activation through G-protein transduction. Also, the natural compounds inhibit PAF-induced neutrophil chemotaxis. It was proved that ginkgolides significantly prolong the average survival time of acute pancreatitis and reduce mortality of the patients. Following pancreatitis, some patients develop an infection. However, in this case, ginkgolides may also be beneficial by possessing inhibitory activity against gram-positive and gram-negative bacteria. Administration of ginkgolides prevents bacterial dissemination—ginkgolides inhibit bacterial shift to the pancreas. The substance with BN52021 chemical code, which is an extract from the *G. biloba* tree, is one of the most promising PAFR antagonists applied in acute pancreatitis [41].

### Ischemia

Myocardial infarction, stroke, and peripheral vascular disease are included in ischemia disorders. Ischemia disorders are one of the most common death cases. Only in a recent century, patients with early signs of myocardial infarction almost always presented thrombotic occlusion of the artery, responsible for the supply of the infected region of their hearts [42]. Stress-induced ischemic injury is manifested by the production and release of PAF. Clogged arteries may lead to cerebral insufficiency. Ginkgolides enhanced the local cerebral blood flow. Interestingly, to inhibit the PAF-mediated aggregation of human platelets, the concentration of applied ginkgolides must be excessive. Namely, in vivo studies show at least 200 times higher numbers than normal rabbit cells. The treatment of BN 52,021 (25 mg/kg/dose, two serial doses) decreased cerebral infarction incidence to 30% [35].

In China, the diterpene ginkgolides meglumine injection (DGMI) is clinically used to treat ischemic stroke. The main components of DGMI are ginkgolides A, B, and C. DGMI was proven to significantly reduce infarct volumes and neurological deficit scores of rats with induced acute cerebral ischemic injury. Furthermore, ginkgolides were proven to inhibit neuronal apoptosis by inducing protein kinase B (Akt) phosphorylation, which affects the Akt/Nrf2 (nuclear factor-erythroid 2-related factor 2), and Akt/CREB (cyclic AMP-responsive element-binding protein) pathways. Besides, it also activates PI3K/AKT (Phosphoinositide 3-kinase) pathway [43].

Cerebral ischemia is manifested by a breakdown of the blood–brain barrier (BBB), which is essential for maintaining a suitable CNS environment, protecting from pathogens and toxins. When the components of BBB (tight junctions, astrocytes, pericytes, basement membrane) are disrupted, a hemorrhage in ischemic cerebral tissue may occur. Treatment with ginkgolides reduces endothelial permeability and upregulates tight junction proteins' expression, leading to reduced BBB damage [44].

### Alzheimer’s disease

Alzheimer's disease (AD) is one of the most prevalent neurodegenerative diseases in which the irreversible loss of neurons occurs. AD symptoms include progressive impairment in memory, judgment, decision making, orientation to physical surroundings, and language. Pathologically AD is characterized by cellular deposits of β-amyloid (Aβ) extracellular plaques and neurofibrillary tangles—constituted of a hyperphosphorylated form of the microtubular protein tau, and neuronal loss [14]. Aβ is a 38 to 43 amino acid peptide derived from the β-amyloid precursor protein (APP) through sequential cleavages by beta- and alpha-secretase enzyme activities [45].

Ginkgolides inhibit the formation of amyloid beta-derived diffusible neurotoxic soluble ligands (ADDLs) in a dose-dependent manner [35]. Besides, they also enhance the activity of alpha-secretase, the enzyme involved in the regulation of the non-amyloidogenic processing of APP. Furthermore, ginkgolides were proven to lower the circulating free cholesterol, which may be involved in the production of APP and Aβ peptide [46]. Aβ was examined to reduce the amount of cell-associated synaptophysin—a transmembrane glycoprotein of presynaptic vesicles. Synaptophysin I (Syp I) contains at least four Syp I subunits and proteins identified as Synaptobrevin. Syp I is phosphorylated on its cytoplasmic transmembrane section by both serine/threonine and tyrosine kinases. Furthermore, Syp I exhibited calcium binding activity. Syp I is a vesicular calcium sensor that takes part in synaptic vesicle biogenesis, the role of Syp I is essential for neurotransmission [47]. The effect of 50 nM Aβ1-42 administration on synapse was the 50% reduction of synaptophysin, indicating the synapse damage. Pre-treatment of ginkgolides A or B protected the cortical and hippocampal neurons from Aβ—induced loss of synaptophysin. The other compound of EGB-761: myricetin and quercetin, did not exhibit analogical properties. Interestingly, other PAF antagonists [48] also reduced the loss of synaptophysin. PAF was proven to mimic the effect of Aβ and cause a reduction of synaptophysin. However, the ginkgolides do not affect the incorporation of Aβ1-42 into neurons. The mechanism of the protective effect of ginkgolides is still sought. Ginkgolides may bind directly to Aβ1-42 peptides and promote the formation of an inactive conformer. Pre-treatment with Ginkgolide B reduced both Aβ1-42 and PAF-induced production of prostaglandin E2 (PGE2) and did not alter the effects of PGE2. Bate et al. proved that the activation of PLA2 by Aβ1–42 leads to sustained PAF production, increasing synapse-damaging PGE2 production [48]. Treatment with ginkgolides positively affects the age of AD onset. In opposite to what is written in the previous paragraph, oral administration of ginkgolides showed high bioavailability indeed, suggesting that the ginkgolides can cross BBB and penetrate the CNS[48]. However, some studies did not reveal any beneficial effect of the administration of ginkgolides on AD development.

### Parkinson’s disease

Parkinson's disease (PD) is a neurodegenerative disease associated with progressive dopaminergic neuron degeneration, accumulation of alfa-synuclein within the substantia nigra pars compacta, and development of Lewy bodies [49]. PD symptoms include significant motor abnormalities, postural instability, tremors, and rigidity—likewise, nonmotor symptoms comprising cognitive impairment, psychiatric symptoms, and sleep disorders. Treatment of PD focuses on increasing dopamine concentration or decreasing the excitability of cholinergic neurons. In some cases, treatment with dopamine agonists might leads to life-threatening complications. Herbal medicine with fewer side effects and higher efficiency may be an alternative therapy for PD [50]. Studies have proven that ginkgolides' neuroprotective effect influences the PD models by increasing cell activity, decreasing mitochondrial dysfunction, inhibiting ROS activation, and limiting apoptosis [51]. Furthermore, Ginkgolide B disrupts tyrosine hydroxylase (TH) degeneration, a rate-limiting enzyme responsible for synthesizing the neurotransmitters dopamine and catechol. The decrease in TH level is reflected by a decrease in the number of dopamine neurons. In the PD model treated by Ginkgolide B, immunohistochemical analysis showed that TH expression was significantly increased. Ginkgolide B achieves very poor water solubility, and oral administration exhibits low brain penetration and blood bioavailability. As for now, one of the improvements of oral absorption of ginkgolide B is an encapsulation of Ginkgolide B [52]. Interestingly other than EGB 761 Biloba extracts like 2 G or GBDP show even better effects against PD. 2 G was reported to induce reversible inhibition of mono-amino oxidase A and B (MAO-A, MAO-B). MAO isoforms regulate levels of biogenic amines in the brain (including dopamine). The level of MAO-B activity increase with age; thus MAO-B inhibitors improve the quality of life of older people [35].

## Conclusions

Ginkgolides show effects adequate for neurotransmitters—by binding with ionotropic receptors and for neuromodulators—by binding to the metabotropic receptors. Besides, the natural compounds regulate a vast spectrum of neurotransmitters (glutamate, gamma-aminobutyric acid, serotonin). It takes time to saturate receptors with ginkgolides; thus, for the clinical application for the successful regulation of the metabolic pathways, the administration of ginkgolides must be long-term. Ginkgolides exhibit a broad and diverse array of neuro-regulatory properties, which still require further investigation. However, further research should focus on overcoming ginkgolides' disadvantages and widening their clinical applications.

## References

[1] Isah T (2015). Rethinking *Ginkgo biloba* L.: medicinal uses and conservation. Pharmacogn Rev.

[2] Bodalski T, Katarzyna K-B (2006). *Ginkgo biloba*—miłorząb dwuklapowy (chemizm i działanie biologiczne) (*Ginkgo biloba*—(chemistry and biological action)). Postępy Fitoter.

[3] Elliott GR, Barchas JD (1980). Changing concepts about neuroregulation: neurotransmitters and neuromodulators. Hormones and the brain.

[4] Herlenius E, Lagercrantz H (2001). Neurotransmitters and neuromodulators during early human development. Early Hum Dev.

[5] Wyllie DJA, Chen PE (2007). Taking the time to study competitive antagonism. Br J Pharmacol.

[6] Chen L, Deng H, Cui H (2018). Inflammatory responses and inflammation-associated diseases in organs. Oncotarget.

[7] McManus LM, Pinckard RN (2000). PAF, a putative mediator of oral inflammation. Crit Rev Oral Biol Med.

[8] Li C, Liu K, Liu S (2020). Role of ginkgolides in the inflammatory immune response of neurological diseases: a review of current literatures. Front Syst Neurosci.

[9] Ishii S, Shimizu T (2000). Platelet-activating factor (PAF) receptor and genetically engineered PAF receptor mutant mice. Prog Lipid Res.

[10] Notarangelo LD (2013). Functional T cell immunodeficiencies (with T cells present). Annu Rev Immunol.

[11] Gandhi CR, Olson MS (1991). PAF effects on transmembrane signaling pathways in rat kupffer cells. Lipids.

[12] Gui C, Zhu W, Chen G (2007). Understanding the regulation mechanisms of PAF receptor by agonists and antagonists: molecular modeling and molecular dynamics simulation studies. Proteins Struct Funct Genet.

[13] Xia SH, Fang DC (2007). Pharmacological action and mechanisms of ginkgolide B. Chin Med J (Engl).

[14] Nussbaum RL, Ellis CE (2003). Alzheimer’s disease and Parkinson’s disease. N Engl J Med.

[15] Li X, Qin J (2005). Modulation of Toll-interleukin 1 receptor mediated signaling. J Mol Med.

[16] Chen K, Sun W, Jiang Y (2017). Ginkgolide B suppresses TLR4-mediated inflammatory response by inhibiting the phosphorylation of JAK2/STAT3 and p38 MAPK in high glucose-treated HUVECs. Oxid Med Cell Longev.

[17] Burdan F, Chałas A, Szumiło J (2006). Cyklooksygenaza i prostanoidy—znaczenie biologiczne (cyclooxygenase and prostanoids—biological implications). Postepy Hig Med Dosw.

[18] Bate C, Reid S, Williams A (2004). Phospholipase A2 inhibitors or platelet-activating factor antagonists prevent prion replication. J Biol Chem.

[19] Willard SS, Koochekpour S (2013). Glutamate, glutamate receptors, and downstream signaling pathways. Int J Biol Sci.

[20] Ożarowski M, Mikołajczak PŁ, Kujawski R (2008). The influence of biologically active compounds contained in medicinal plants on the receptors of the central nervous system—the basis of potential mechanisms of interaction with synthetic drugs. Part 2. Herba Pol.

[21] Chatterjee SS, Kondratskaya EL, Krishtal OA (2003). Structure-activity studies with *Ginkgo biloba* extract constituents as receptor-gated chloride channel blockers and modulators. Pharmacopsychiatry.

[22] Heads JA, Hawthorne RL, Lynagh T, Lynch JW (2008). Structure-activity analysis of ginkgolide binding in the glycine receptor pore. J Neurochem.

[23] Goudet C, Magnaghi V, Landry M (2009). Metabotropic receptors for glutamate and GABA in pain. Brain Res Rev.

[24] Watanabe M, Maemura K, Kanbara K (2002). GABA and GABA receptors in the central nervous system and other organs. International review of cytology.

[25] Thompson AJ, Lester HA, Lummis SCR (2010). The structural basis of function in Cys-loop receptors. Q Rev Biophys.

[26] Huang SH, Lewis TM, Lummis SCR (2012). Mixed antagonistic effects of the ginkgolides at recombinant human ρ1 GABAC receptors. Neuropharmacology.

[27] Thompson AJ, Lummis SCR (2009). Calcium modulation of 5-HT3 receptor binding and function. Neuropharmacology.

[28] Hornykiewicz O (1966). Dopamine (3-hydroxytyramine) and brain function. Pharmacol Rev.

[29] Juárez Olguín H, Calderón Guzmán D, Hernández García E, Barragán Mejía G (2016). The role of dopamine and its dysfunction as a consequence of oxidative stress. Oxid Med Cell Longev.

[30] Yoshitake T, Yoshitake S, Kehr J (2010). The *Ginkgo biloba* extract EGb 761® and its main constituent flavonoids and ginkgolides increase extracellular dopamine levels in the rat prefrontal cortex: research paper. Br J Pharmacol.

[31] Yeh KY, Wu CH, Tai MY, Tsai YF (2011). *Ginkgo biloba* extract enhances noncontact erection in rats: the role of dopamine in the paraventricular nucleus and the mesolimbic system. Neuroscience.

[32] Guo M, Suo Y, Gao Q (2015). The protective mechanism of ginkgolides and *Ginkgo* flavonoids on the TNF-α induced apoptosis of rat hippocampal neurons and its mechanisms in vitro. Heliyon.

[33] Chan WH (2007). The signaling cascades of ginkgolide B-induced apoptosis in MCF-7 breast cancer cells. Int J Mol Sci.

[34] Murray RK, Granner DK, Rodwell VW (2016). Harpers ilustrated biochemistry.

[35] Omar SH (2013). Ginkgolides and neuroprotective effects. Natural products: phytochemistry, botany and metabolism of alkaloids, phenolics and terpenes.

[36] Liu Q, Jin Z, Zhiliang Xu, Yang H, Li L, Li G, Li F, Shaoli Gu, Zong S, Zhou J, Cao L, Zhenzhong Wang WX (2019). Antioxidant effects of ginkgolides and bilobalide against cerebral ischemia injury by activating the Akt/Nrf2 pathway in vitro and in vivo. Natl Libr Med.

[37] Scholtyssek H, Damerau W, Wessel R, Schimke I (1997). Antioxidative activity of ginkgolides against superoxide in an aprotic environment. Chem Biol Interact.

[38] Taylor JB, Triggle DJ (2007). Comprehensive medicinal chemistry II.

[39] Sarkar C, Quispe C, Jamaddar S (2020). Therapeutic promises of ginkgolide A: a literature-based review. Biomed Pharmacother.

[40] Leung PS, Ip SP (2006). Pancreatic acinar cell: its role in acute pancreatitis. Int J Biochem Cell Biol.

[41] Chen C, Xia SH, Chen H, Li XH (2008). Therapy for acute pancreatitis with platelet-activating factor receptor antagonists. World J Gastroenterol.

[42] DeWood MA, Spores J, Notske R (1980). Prevalence of total coronary occlusion during the early hours of transmural myocardial infarction. N Engl J Med.

[43] Zhang W, Song JK, Yan R (2018). Diterpene ginkgolides protect against cerebral ischemia/reperfusion damage in rats by activating Nrf2 and CREB through PI3K/Akt signaling. Acta Pharmacol Sin.

[44] Feng Z, Sun Q, Chen W (2019). The neuroprotective mechanisms of ginkgolides and bilobalide in cerebral ischemic injury: a literature review. Mol Med.

[45] Chow VW, Mattson MP, Wong PC, Gleichmann M (2010). An overview of APP processing enzymes and products. Neuromol Med.

[46] Yao ZX, Han Z, Drieu K, Papadopoulos V (2004). *Ginkgo biloba* extract (Egb 761) inhibits β-amyloid production by lowering free cholesterol levels. J Nutr Biochem.

[47] Mcmahon HT, Bolshakov VY, Janz R (1996). Synaptophysin, a major synaptic vesicle protein, is not essential for neurotransmitter release. Proc Natl Acad Sci USA.

[48] Bate C, Tayebi M, Williams A (2008). Ginkgolides protect against amyloid-β1–42-mediated synapse damage in vitro. Mol Neurodegener.

[49] Maiti P, Manna J, Dunbar GL (2017). Current understanding of the molecular mechanisms in Parkinson’s disease: targets for potential treatments. Transl Neurodegener.

[50] Yu D, Zhang P, Li J (2021). Neuroprotective effects of *Ginkgo biloba* dropping pills in Parkinson’s disease. J Pharm Anal.

[51] Wu T, Fang X, Xu J (2020). Synergistic effects of ginkgolide B and protocatechuic acid on the treatment of Parkinson’s disease. Molecules.

[52] Zhao Y, Xiong S, Liu P (2020). Polymeric nanoparticles-based brain delivery with improved therapeutic efficacy of ginkgolide B in Parkinson’s disease. Int J Nanomed.

